# The functional and psychological impact of delayed hip and knee arthroplasty: a systematic review and meta-analysis of 89,996 patients

**DOI:** 10.1038/s41598-024-58050-6

**Published:** 2024-04-05

**Authors:** G. M. Cooper, J. M. Bayram, N. D. Clement

**Affiliations:** 1 University of Edinburgh Medical School, 47 Little France Crescent, Edinburgh, EH16 4TJ UK; 2 Edinburgh Orthopaedics, Royal Infirmary of Edinburgh, 51 Little France Crescent, Edinburgh, EH16 4SA UK

**Keywords:** Outcomes research, Rheumatic diseases, Health services, Quality of life, Rheumatology

## Abstract

This systematic review and meta-analysis aimed to determine the impact of presurgical waiting times on pre-/post-operative joint specific pain and function, health-related quality of life (HRQOL) and perspectives of patients awaiting primary elective total hip (THR) and knee (TKR) replacements. MEDLINE, EMBASE, PUBMED, and CENTRAL databases were searched from inception until 30th January 2023 (CRD42022288128). Secondary literature and unpublished datasets containing paediatric, non-elective, partial, or revision replacement populations were excluded. PRISMA 2020 reporting and GRADE certainty of evidence guidelines were followed. Residual maximum likelihood meta-analysis and linear meta-regression was performed to elucidate the influence of presurgical waiting time. Twenty-six studies were eligible for systematic review and sixteen for meta-analysis, capturing 89,996 patients (60.6% female, mean age 67.4 years) between 2001 and 2022. A significant deterioration in joint function (mean difference (MD):0.0575%; 95% CI 0.0064, 0.1086; *p* = 0.028(4d.p.); I2 = 73.1%) and HRQOL (MD: 0.05%; 95% CI − 0.0001.0009; *p* = 0.011(4 d.p.); I2 = 80.6%) was identified per additional day of waiting. Despite qualitative evidence, meta-analysis could not observe a relationship with postoperative outcome data. Patient responses to delayed THR and TKR surgery were unanimously negative. Immediate action should seek to reduce the increased patient anxiety and significant reductions in pre-operative joint functionality and HRQOL associated with prolonged pre-surgical waiting time, whilst mitigating any potential deleterious post-operative effects.

## Introduction

Primary total hip (THR) and knee replacements (TKR) are amongst the most common elective orthopaedic procedures^1^. In the UK, the mortality-adjusted lifetime risk for a THR and TKR at fifty years-old is 11.6% and 10.8% and 7.1% and 8.1% for females and males, respectively, whilst, in the USA, over 50% of patients diagnosed with symptomatic knee osteoarthritis will undergo TKR^2,3^. Most frequently, these surgeries are performed in the management of end-stage osteoarthritis—a degenerative process which accounts for 2% of global disability years, for example impacting 10% of UK adults^4,5^.

Following the COVID-19 pandemic, the total number of THRs and TKRs performed in the UK has halved and not yet recovered^6,7^. Concurrently, demand has continued to rise, resulting in extended waiting lists^6,7^. Similar trends can be seen in comparable health systems including, Canada, the Netherlands, and Denmark^8,9^. Consequently, understanding how increased waiting-times for elective primary THR/TKR influences patient-centred outcomes will be essential for future healthcare planning.

Previously a systematic review of 1,646 patients with osteoarthritis awaiting THR and TKR by Hoogeboom et al. identified no evidence of deterioration in self-reported pain status^10^. However, this analysis was limited to the first 180 days of waiting for THR or TKR^10^. More recently, Patten et al. concluded that the pain levels of patients with osteoarthritis remained stable for the first year after addition to a surgical waiting list, although only reported a median follow-up of 13.6 weeks^11^. Furthermore, both reviews did not measure changes in joint functionality, quality of life, wider patient perceptions, nor explore how prolonged pre-surgical waiting time might impact postoperative outcomes.

To better inform elective service planning, this systematic review and meta-analysis sought to understand how pre-surgical waiting time—defined as the time from placement on surgical waiting list until surgery—for patients undergoing elective primary THR or TKR influenced both pre- and post-operative joint specific pain and functional status, global health-related quality of life (HRQOL), and patient perspectives.

## Results

### Literature summary and evaluation

Study selection is summarised in Supplementary Fig. 1. After deduplication, 525 studies were initially screened for eligibility with substantial inter-rater reliability (k = 0.75)^12^. The remaining thirty-four studies were then assessed in-detail, with twenty-six being included within this review^13–38^. Subsequently, sixteen studies were suitable for quantitative meta-analysis. Exclusory causes and summary characteristics for eight fully assessed studies are summarised in Supplementary Table 2^39–46^.

The included studies capture a reported population of 89,996 patients (60.6% female, mean age 67.4 years) between 2001–2022 (Table 1)^13–38^. Similar study composition and methodologies enabled valid comparisons. Individual patient meta-analysis was not feasible. Risk of bias for each extracted study outcome for randomised controlled trial, non-randomised (case–control and cohort), and cross-sectional studies are summarised in Supplementary Tables 3, 4, and 5 with the ROB-2, ROBINS-I, and JBI frameworks, respectively^47–49^. Supplementary Table 6 presents Grading of Recommendations Assessment, Development and Evaluation (GRADE) outcome certainty evaluations^50^.

**Table 1:** Summary characteristics of included studies.

Authors	Study design	Study setting				Study population in analysis				Study outcomes
		Eligibility criteria	Study period	Country	Number of centres	Grouped mean waiting time (Days)	Sample size [WT groups]	Mean age (years) [WT groups]	Sex (% female) [WT groups]	
Kelly et al.^13^	Prospective cohort	Patients waiting for > 1 month for THR/KR	1995–1997	Canada	2 Hospitals	132	313	68.1	59%	Disease symptoms, global HR-QOL
Hajat et al.^15^	Prospective cohort	Patients undergoing THR	1996–1997	UK	143 Hospitals	< 46, 46–183, 183–365, > 365	7,151 [2,316; 1,120; 453; 203]	69.6*[N/A]	58% [N/A]	Hip function
Mahon et al.^14^	Prospective cohort	Patients undergoing THR for OA	1994–1997	Canada	Single-Centre	37, 329	99 [63;36]	68.0 [N/A]	51% [N/A]	Disease symptoms, global HR-QOL, patient anxiety
Nilsdotter and Loh-mander^16^	Prospective cohort	Consecutive patients aged > 50 under-going unilateral THR	1997–1998	Sweden	Single-Centre	61, 155	56 [N/A]	72.0 [N/A]	57% [N/A]	Disease symptoms, global HR-QOL
Ostendorf et al.^17^	Prospective cohort	Patients waiting for THR	1999–2000	Netherlands	3 Hospitals	183	161	68.4	66%	Hip function, disease symptoms, global HR-QOL
Fielden et al.^18^	Prospective cohort	Patients aged > 20 awaiting primary THR due to OA	1999–2002	New Zealand	4 Hospitals	155	153	66.0	65%	Disease symptoms, global HR-QOL, financial burden of disease
Garbuz et al.^20^	Prospective cohort	Patients undergoing primary THR for OA	2001–2003	Canada	Single-Centre	183	147	65.0	56%	Disease symptoms
Hirvonen et al.^19^	Prospective cohort	Patients awaiting primary TH/KR for OA; matched population controls	2002–2003	Finland	3 Hospitals	71*	266	67.6	82%	Global HR-QOL
Ahmad and Konduru^23^	Prospective cohort	Patients undergoing primary THR for OA	2003–2004	UK	Single-Centre	242	58	68.7	51.7%	Hip function
Hirvonen et al.^22^	RCT	Patients awaiting primary TKR > 16 years-old	2002–2003	Finland	3 Hospitals	73, 266	310 [127, 183]	67.7 [66.0, 69.0]	68.7% [70.1%, 67.8%]	Knee function and global HR-QOL
Kapstad et al.^23^	Prospective cohort	Norwegian-speaking patients aged > 18 years-old who had waited > 30 days for TH/KR	2003–2004	Norway	3 Hospitals	71, 102	170 [N/A]	67.9 [N/A]	72.9% [N/A]	Disease symptoms
McHugh et al.^24^	Prospective cohort	Patients awaiting primary THR/KR	2003	UK	Single-Centre	91	84	68.0*	59.0%	Global HR-QOL, disease symptoms and pain progression
Escobar et al.^25^	Prospective cohort	Consecutive patients waiting primary TH/KR for OA	2003–2004	Spain	6 Hospitals	201	684	70.1	62.0%	Disease symptoms
Desmeules et al.^26^	Prospective cohort	Consecutive, French-speaking, insured, patients > 40 years-old at 3 hospitals awaiting primary TKR	2006–2007	Canada	3 Hospitals	183	153	66.0	65.0%	Disease symptoms, global HR-QOL
Tuominen et al.^27^	RCT	Patients awaiting primary TKR for OA > 16 years-old	2002–2003	Finland	3 Hospitals	95, 239	330 [132, 198]	67.6 [67.0, 68.0]	71.8% [74.2%, 70.2%]	Knee function, global HR-QOL, healthcare-economic costs of delayed treatment
Desmeules et al.^28^	Prospective cohort	Consecutive, French-speaking, insured, patients > 40 years-old at 3 hospitals awaiting primary TKR	2006–2007	Canada	3 Hospitals	184	141	66.0	66.0%	Disease symptoms, global HR-QOL
Skou et al.^29^	RCT	Randomised to TKR and physiotherapy	2011–2013	Denmark	Single-Centre	31	50	65.8	64.0	Knee function; global HR-QOL
Nikolova et al.^30^	Retrospective cohort	Patients undergoing elective THR/TKR within NHS England	2009–2010	UK	Multicentre (all of NHS England)	78, 79	61,905	69.0	60%	Hip function, global HR-QOL, healthcare-economic costs of delayed treatment
Brown et al.^31^	Cross sectional	Patients awaiting primary elective TH/KR	2020	USA	15 Hospitals	N/A	848	62.6	56.6	Emotional impact of delay, pain and symptom progression
Clement et al.^32^	case–control	Patients awaiting TH/KR was controlled against un-matched previously published dataset	2014–2017; 2020	UK	10 Hospitals	365, 91.3	5,084 [843, 4241]	68.5 [69.1, 68.4]	58.9 [59.2, 58.8]	Global HR-QOL, willingness and perception to undergoing surgery
Farrow et al.^33^	Case- control	Patients awaiting primary TH/KR	2020	UK	Single-Centre	365* (Control); 455* (COVID-19)	548 [260, 288]	69 [69. 68]	58.2 [54.6, 61.5]	Duration of waiting time to follow-up, analgesic prescription
Johnson et al.^34^	Cross sectional	Patients awaiting elective hip or knee arthroplasty*	2020	USA	Single-Centre	N/A	113	N/A	N/A	Duration of delay, Emotional impact of delay, Pain progression
Clement et al.^35^	Cross sectional	Patients awaiting primary TH/KR	2021–2022	UK	4 Hospitals	182.5	326	68.6	54.0	Global HR-QOL
Holzapfel et al.^37^	Retrospective cohort	Patients undergoing primary TH/KR	2011–2020	Germany	Single-Centre	< 1; > 1	10,140 [7760; 2480]	66.1 [66.1; 66.0]	57.6 [57.2; 58.9]	Postoperative complications
Grace et al.^36^	Cross sectional	Patients awaiting primary TH/KR	N/A (3-months)	USA	Single-Centre	N/A	200	66.0	53.0	Participation in physiotherapy
Morri et al.^38^	Retrospective cohort	Patients admitted for primary TH/KR	2020	Italy	Single-Centre	N/A [Control; COVID-19]	463 [183; 280]	66.0 [65.8; 66.1]	44.9 [43.2; 46.1]	Hip/knee function

Summary characteristics of studies included in systematic review and meta-analysis: WT: Waiting time; THR: Total Hip Replacement; OA: Osteoarthritis; N/A: Not Available; HR-QOL: Health-Related Quality of Life; TKR: Total Knee Replacement; TH/KR: Total Hip/ Knee Replacement; RCT: Randomised Controlled Trial; NHS: National Health Service (UK). *Median Value.

Reported joint-specific pain and function and HRQOL outcome data with a preoperative endpoint are qualitatively summarised in Table 2. Comparatively, the studies presented in Table 3 show joint-specific pain and function and HRQOL data postoperatively. Table 4 outlines patient perspectives on prolonged presurgical waiting time for primary elective THR and THR.

**Table 2:** Summary of findings from studies reporting changes in clinical functionality and health-related quality of life between baseline and preoperative endpoint.

Study	Analysis type	Mean waiting time (days)	Outcome (outcome measure[s])	
			Joint-specific outcome score	Health-related quality of life index score
Kelly et al.^13^	UVR, MVR	132	**No significant changes in all WOMAC subdomains over the course of the waiting time**	***UVR: Improvement in THR SF-36 BP, ER, MH & GH (3.5, 15.9, 3.1, 3.2%) & TKR SF-36 GH (3.6%) over the course of the waiting time. No other significant comparisons***
Nilsdotter and Lohmander^16^	MD	61, 155	***Preoperative WOMAC pain score improved when waiting less than 3 months for surgery. No other significant comparisons***	***Preoperative SF-36 GH improved for patients waiting time greater than 3 months. No other significant comparisons***
Ostendorf et al.^17^	MD	183	*There was a 4.5% deterioration in WOMAC pain, 3.5% in WOMAC function and 4.4% in OHS whilst awaiting surgery*	*SF-36 recorded 3.5% and 3.8% reductions in BP and PF subdomains, respectively, whilst awaiting surgery*
Hirvonen et al.^19^	UVR (Linear)	71*	N/A	**No significant change in 15D**
Ahmad and Konduru^21^	UVR (Linear)	242	*Deteriorations in OHS whilst awaiting surgery was associated with waiting time in males, females, younger and older patients (10.0, 9.7, 12.2, 9.0%/ 6 months)*	N/A
Hirvonen et al.^22^	MD (Intention to Treat)	73, 266	N/A	**No significant difference in 15D**
Kapstad et al.^23^	MD, MVR (Linear)	71, 102	*TKR patients deteriorated by 3% in WOMAC Physical Function (MD) only, whilst awaiting surgery*	N/A
McHugh et al.^24^	MD	91	*Across the whole study group whilst awaiting surgery:* *VAS pain deteriorated between baseline to 3 & 6 months (6 & 12%)* *WOMAC pain deteriorated between baseline and 3 months (6%)* *WOMAC physical function deteriorated between baseline to 3 & 6 months (7.1 & 4.3%)*	***SF-36 GH and RE subdomains improved by 7.9 and 8.5%, respectively between baseline and 6 months***
Desmeules et al.^26^	MD	183	*The whole study group experienced a decline in WOMAC pain, function, and contralateral knee pain (2.8, 4.6, 4.7%), whilst awaiting surgery*	*The whole study group SF-36 physical function deteriorated by 4.8%, whilst awaiting surgery*
Tuominen et al.^27^	MD	95, 239	**No significant changes were observed in KSCRS Pain or Functional scores whilst awaiting surgery**	**No significant changes were observed in 15D index whilst awaiting surgery**
Brown et al.^31^	Descriptive	Increased*	**489 of 848 patients reported increased pain since the beginning of the COVID-19 pandemic**	N/A
Clement et al.^32^	MVR (Linear)	274	N/A	*Each additional month of waiting was associated with a 1.35% reduction in EQ-5D quality of life*
Farrow et al.^33^	MVR	Control: 365*, COVID-19: 455*	*MVR OR of being on any opioid at presurgical follow-up was 1.84 between control and COVID-19 cohort (longer waiting time only reported confounder)*	
Johnson et al.^34^	Descriptive	Increased*	**68 of 117 patients reported increased pain with increased waiting time**	N/A
Clement et al.^35^	MD	182.5	N/A	*Patients awaiting primary elective THR and TKR reported a mean decline of 0.175, relative to their HR-QOL status 6 months previously (from 0.492 to 0.317, EQ-5D)*

Summary of preoperative reported findings. Bold, italics, bolditalics indicates significant findings pertaining to the impact of waiting time on reported outcomes. Bolditalics: indicates a significant positive outcome correlated with increased waiting period; bold, a non-significant finding; italics: a significant negative outcome correlated with increased waiting period. Where mean differences were provided they were converted into a percentage change. *Median (Range); UVR: Univariate Regression; MVR: Multivariate Regression; MD: Mean Difference; OR: Odds Ratio; OHS: Oxford Hip Score; HHS: Harris Hip Score; OKS: Oxford Hip Score (12–60 best-to-worst); KSCRS: Knee Society Clinical Rating System; WOMAC: Western Ontario and McMasters Universities Osteoarthritis Index (0–100, worst-to-best); HR-QOL: Health-Related Quality of Life; SF-36 (0–100, worst-to-best): Short Form 36-Item- Subdomains: Bodily Pain (BP), Physical Function (PF), Role Physical (RP), Role Emotional (RE), Mental Health (MH), Vitality (VT), Social Function (SF), General Health (GH); EQ-5D: European Quality of Life 5-Dimensional Index; 15D: 15-Dimension Score Sheet; EQ-VAS: European Quality of Life Visual Analogue Scale; (Pain) VAS: Visual Analogue Scale of Pain; THR: Total Hip Replacement; TKR: Total Knee Replacement. *Whilst waiting time was not quantified, these studies followed patients where initial surgery had been cancelled due to the COVID-19 pandemic.

**Table 3:** Summary of findings from studies reporting clinical functionality and health-related quality of life with a postoperative endpoint.

Study	Analysis type	Mean waiting time (days)	Follow-up duration (days)	Outcome (outcome measure[s])	
				Joint-specific outcome score	Health-related quality of life index score
Hajat et al.^15^	MD, MVR (Linear)	46, 152, 289, 365	365	Estimated decline in OHS in outpatient wait 6–12 (2.6%) or > 6 months (5.4%) and inpatient wait 6–12 (1.7%), 12–18 (3.5%) and > 18 months (3.3%)	N/A
Mahon et al.^14^	MD	37,329	90	Longer wait time was associated with reduced postoperative improvement in all WOMAC domains (28% compared to 41% improvement)	Patients waiting longer experienced reduced gains in SF-36 bodily pain (29.3% vs 18% improvement), physical function (25.7% vs 14.4% improvement) and state anxiety subdomains*
Nilsdotter and Lohmander^16^	MD	61, 155	365	Postoperative WOMAC score did not statistically differ between inpatient waiting time less or greater than 3 months	Postoperative SF-36 score did statistically differ between inpatient waiting time less or greater than 3 months
Ostendorf et al.^17^	MD, MVR (Correlation)	183	365	Length of waiting time was the only predictor for deterioration in OHS, WOMAC Pain, and WOMAC Function (R2 = 0.7, 0.5, 0.3 respectively)	N/A
Fielden et al.^18^	Logistic Regression	155	182.5	No statistically significant association between WOMAC score and waiting time	No statistically significant association between EQ-5D score and waiting time
Garbuz et al.^20^	MVR (logistic)	183	365	Each additional month of waiting was associated with an OR of 0.92 in achieving a “better than expected” WOMAC functional score	N/A
Escobar^25^	UVR (Correlation)	201	182.5	Time on waiting list correlated negatively with WOMAC Pain and Stiffness subdomains (R = -0.09, − 0.11)	N/A
Tuominen et al.^27^	MD	95, 239	365	There was no significant change in KSCRS pain or function subdomains	There was no significant difference in change in 15D score
Desmeules et al.^28^	MD, MVR	184	182.5	Final contralateral knee pain was least in the 3–6 month waiting time group (86.1%) and worst in > 9 months waiting time.*	SF-36 was significantly reduced in the RP domain in the > 9 month waiting time group relat1. BOA. T&O waiting list the largest for over a decade [Internet]. [cited 2024 Feb 14]. Available from: https://www.boa.ac.uk/resource/t-o-waiting-list-the-largest-for-over-a-decade.html ive to the others.*
Skou et al.^29^	MD	0–30*	182.5	Only data from the TKR arm was synthesised for meta-analysis. No direct comparator precludes any conclusions from this study, relevant to this review’s research question	
Nikolova et al.^30^	MVR	THR: 78, TKR: 79	182.5	Each additional week of waiting time corresponded to a − 0.1% reduction in OHS and − 0.04% in OKS	Each additional week of waiting time corresponded with deterioration in EQ-VAS and EQ-5D. In TKR this coefficient was − 0.06% for both and in THR this was − 0.06% and − 0.04%, respectively
Holzapfel et al.^37^	UVR, MVR	13.5	90	Compared with on-time surgery, postponing elective TKR/ THR was associated with an increased risk of revision < 60 (164/2480; 300/7660)and < 90 days (175/2480; 345/7660)	N/A
Morri et al.^38^	UVR, MVR (Logistic)	Increased**	6.9***	Between the pre-COVID-19 and COVID-19 cohorts, there was no significant difference between patients mean time to walk (with antebrachial device) or to climb three stairs	N/A

Summary of postoperative reported findings. Bold, italics indicates significant findings pertaining to the impact of waiting time on reported outcomes. Bold, a non-significant finding; italics: a significant negative outcome correlated with increased waiting period. Where mean differences were provided, they were converted into a percentage change. *Magnitude of change not reported; UVR: Univariate Regression; MVR: Multivariate Regression; MD: Mean Difference; OR: Odds Ratio; OHS: Oxford Hip Score; HHS: Harris Hip Score; OKS: Oxford Hip Score (12–60 best-to-worst); KSCRS: Knee Society Clinical Rating System; WOMAC: Western Ontario and McMasters Universities Osteoarthritis Index (0–100, worst-to-best); HR-QOL: Health-Related Quality of Life; SF-36 (0–100, worst-to-best): Short Form 36-Item- Subdomains: Bodily Pain (BP), Physical Function (PF), Role Physical (RP), Role Emotional (RE), Mental Health (MH), Vitality (VT), Social Function (SF), General Health (GH); EQ-5D: European Quality of Life 5-Dimensional Index; 15D: 15-Dimension Score Sheet; EQ-VAS: European Quality of Life Visual Analogue Scale; (Pain) VAS: Visual Analogue Scale of Pain; THR: Total Hip Replacement; TKR: Total Knee Replacement.*Range. **Whilst waiting time was not quantified, these studies followed patients where initial surgery had been cancelled due to the COVID-19 pandemic.***Patients only followed-up whilst in hospital.

**Table 4:** Summary of findings from studies exploring patient perspectives on prolonged waiting times.

Authors	Time point	Outcome	
		Emotional response to delay	Patient perceptions on elective surgery services
Mahon et al.^14^	Postoperative	Patient anxiety (State-Trait Anxiety Score) showed a significant and greater reduction from baseline (outpatient wait) to post-surgery in patients with an inpatient wait < 6 months, relative to > 6 months. This finding was correlated with duration of inpatient waiting time in < 3; 3–6; 6–12; > 12 months subgroups Patient anxiety levels were elevated during the inpatient waiting period, relative to baseline	N/A
Brown et al.^31^	Preoperative	The greatest source of patient anxiety was unknown duration of delay, followed by transmitting and developing COVID-19 post-operatively, personal finances and job security	N/A
Clement et al.^32^	Preoperative	N/A	52.3% (n = 441/843) patients would prefer a face-to-face consultation with their surgeon over telephone or video consultation. Similarly, 58.7% (n = 495/843) and 49.7% (n = 419/843) of patients would be willing to have surgery with a different surgeon or at a different hospital, respectively, provided equal waiting times
Johnson et al.^34^	Preoperative	75/113 patients (66%) reported negative emotional consequences of delayed surgery. 85/113 (75%) reported negative effects of delayed surgery. 52% (n = 59/113) of patients expressed frustration at the delay to their procedure: 40% (n = 45/113) anxiety; 39% (n = 44/113) worry; 27% (n = 30/113) restlessness and helplessness; 18% (n = 20/113) depression; 12% (n = 14/113) fear and 11.5% (n = 13/113) anger	N/A
Grace et al.^36^	Preoperative		92.5% (n = 185/200) patients were unwilling to delay elective THR or TKR for mandatory PT. Of these, 44% (n = 81) perceived surgery as inevitable long-term; 29% believed PT would not offer pain relief; 13% imagined PT would worsen their pain; 12% thought that delaying surgery would prolong pain; 2% had another reason

Summary of reported patient perspectives. NHS: National Health Service; TKR: Total Knee Replacement; THR: Total Hip Replacement; PHQ-9: 9-Item Patient Health Questionnaire; OHS: Oxford Hip Score; OKS: Oxford Knee Score; PT: Physiotherapy.

### Changes in joint functionality whilst awaiting total hip or knee replacement surgery

**Figure 1:**
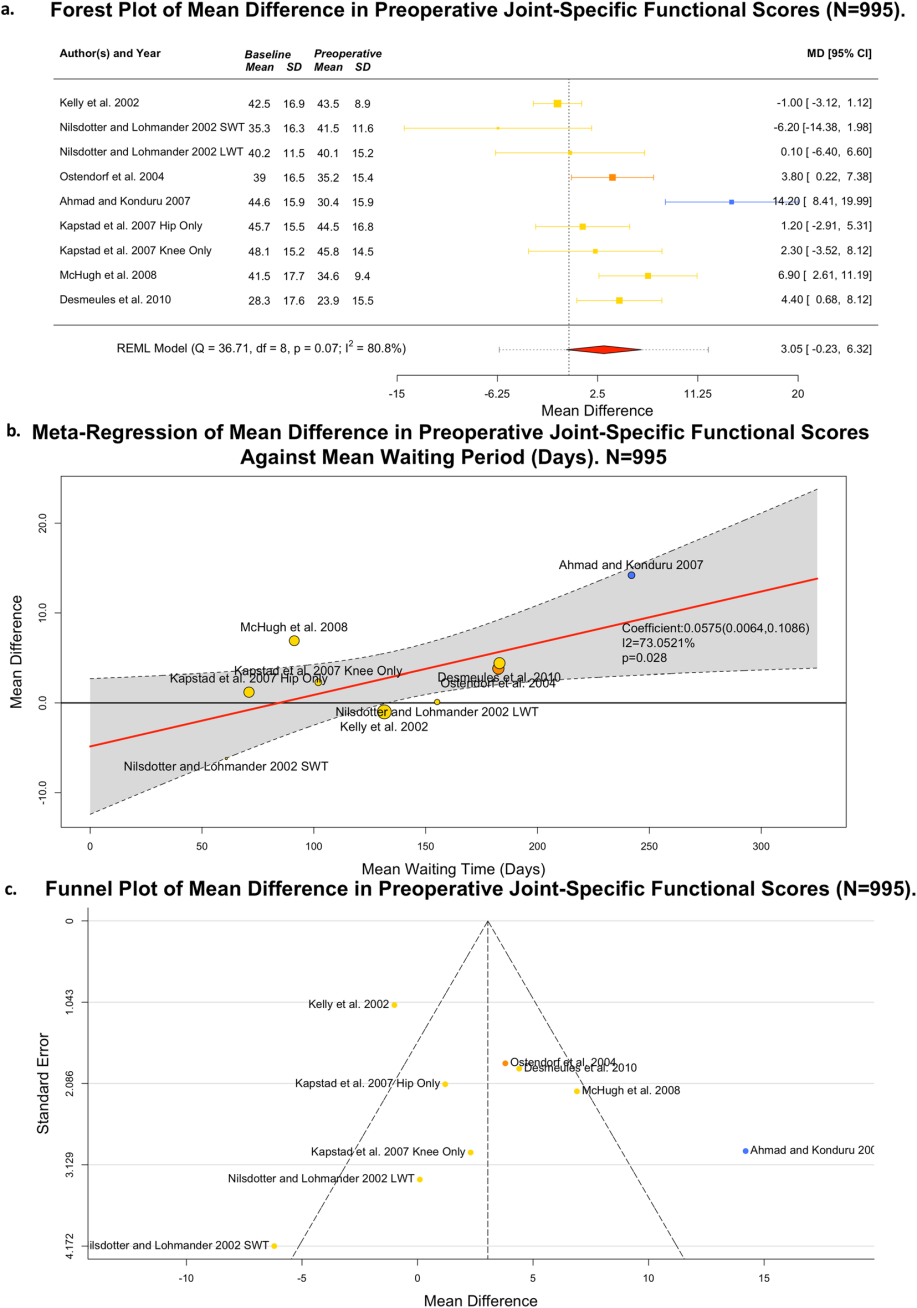
Preoperative Joint-Specific Outcome Data (n = 995]. (**A**) Forest plot of mean difference; (**B**) Meta-regression by mean waiting time; (**C**) Funnel plot. Outcome measures reported include: WOMAC score (Yellow); Oxford Hip Score (Orange) and Oxford Knee Score (Blue].

Fifteen studies commented on deteriorating joint pain/function whilst awaiting THR or TKR, capturing 9,070 patients . Seven of these (n = 995 patients) were suitable for meta-analysis, reporting mean difference changes in joint-specific pain and functional outcomes across nine cohorts of patients awaiting either THR or TKR^13,16,17,21,23,24,26,27,31,33,34^. Figure 1A presents a forest plot comparing changes in joint-specific outcome (0–100, worst-best) whilst awaiting surgery. A deterioration was observed (MD 3.05; 95% CI − 0.32,6.32; *p* = 0.07; I^2^ = 80.8%) between baseline and presurgical scores, which trended towards significance. Consequently, a linear meta-regression was performed (Fig. 1B) to explore the influence of continuous waiting time on this observed heterogeneity, which identified a significant signal corresponding to an absolute deterioration of 0.0575 on a 100-point scale, per additional day of waiting time (95% CI 0.0064,0.1086; *p* = 0.028 (to 4 d.p.); I^2^ = 73.1%).

Tuominen et al. was excluded from this meta-analysis as, despite reporting an acceptable joint-specific outcome (Knee Society Clinical Rating System), the short (n = 132) and non-fixed waiting time (n = 198) groups reported mean differences of zero (to 3 decimal places) after mean respective waits of 94.6 and 239.2 days^27,51^. Given expected random variation in outcome measurement, this introduced concerns of possible reporting error. Both Brown et al. and Johnson et al. were cross-sectional studies and thus unable to identify temporal changes in outcomes. However, in both studies, the majority of patients associated subjective increases in pain with increased waiting times (487/848 and 68/117, respectively)^31,34^.

Farrow et al. reported an odds ratio of 1.84 (95% CI 1.29,2.62; *p* < 0.001) associated with any opioid prescription at presurgical assessment between a 2014–2017 control cohort and a 2020 COVID-19 cohort (median waiting times 365 and 455, respectively)^33^. Although surrogate for both joint functional outcome and HRQOL, opioid prescription is of high clinical relevance to patient and clinician stakeholders and merits the inclusion of this study within qualitative syntheses^52^.

Overall certainty in this outcome was high (Supplementary Table 6). The funnel plot presented in Fig. 1C showed limited risk of publication biases, whilst there was insufficient threat of imprecision, inconsistency, and bias (Supplementary Table 5) to downgrade certainty. Subsequently, this analysis concludes that the observed meta-regression is representative of the true effect and that deterioration in joint function occurs with increasing preoperative waiting period.

### Post-operative impact of waiting time on the joint specific outcome of total hip or knee joint replacement

**Figure 2:**
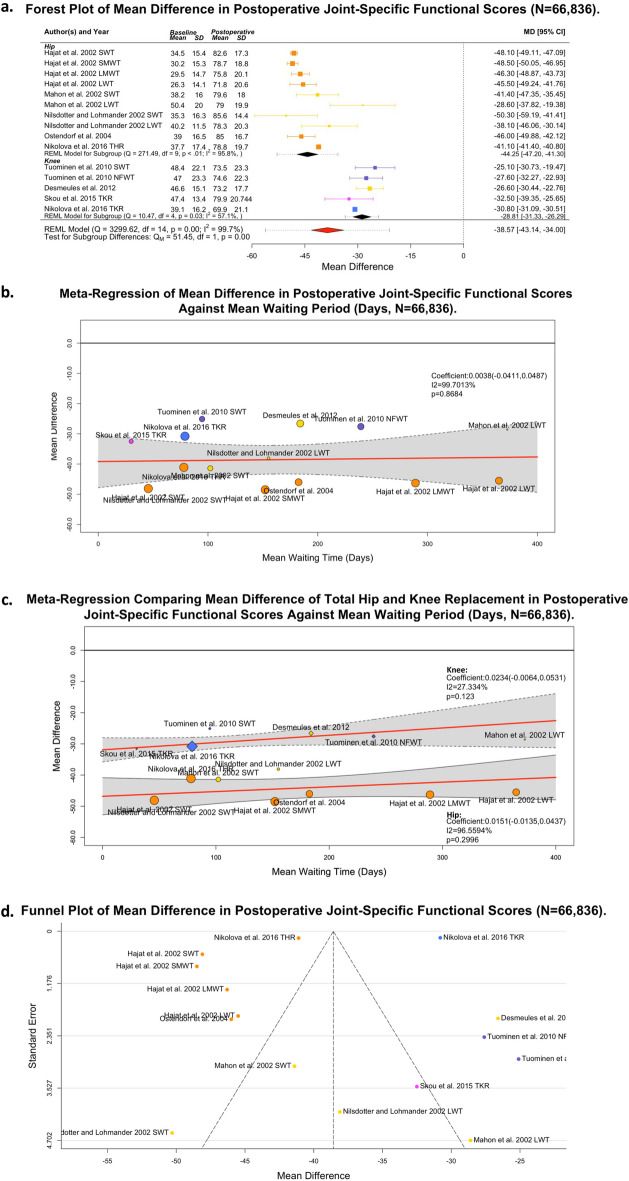
Postoperative Joint-Specific Outcome Data (N = 66,836]. (**A**) Forest plot of mean difference showing Total Hip; (**B**) Meta-regression by mean waiting time; (**C**) Meta-regression sensitivity analysis of Nikolova et al. 2016 by mean waiting time. (N = 4,873); (**D**) Funnel plot. Outcome measures reported included: Western Ontario and McMaster Universities Osteoarthritis Index score (Yellow); Oxford Hip Score (Orange); Oxford Knee Score (Blue), Knee Society Clinical Rating System (Purple), and Knee Injury and Osteoarthritis Outcome Score (Magenta].

Thirteen studies, capturing 81,523 patients, reported on postoperative joint pain and function^14–18,20,25,27–30,37,38^. Fifteen reported cohorts across eight studies were suitable for quantitative meta-analysis (n = 66,836 patients)^14–17,27–30^. Improvement in joint specific outcome was observed postoperatively (MD (0–100 score, worst-to-best) 38.57; 95% CI 34.00,43.14; *p* < 0.001; I^2^ = 99.7%) and presented as a forest plot (Fig. 2A). In the absence of mixed cohorts, it was possible to perform a sensitivity analysis comparing THR and TKR data, which is also shown in Fig. 2A. There was a significantly greater (*p* = 0.000) reported cumulative effect in THR patients (MD 44.25; 95% CI 41.30,47.20; *p* < 0.001; I^2^ = 95.8%), relative to TKR (MD 28.81; 95% CI 26.29,31.33; *p* = 0.003; I^2^ = 57.1%).

To explore how waiting time discriminated this outcome, Fig. 2B presents a pooled linear meta-regression of preoperative waiting time against MD (coefficient 0.00383; 95% CI − 0.0411,0.0487; *p* = 0.8684 (to 4 d.p.); I^2^ = 99.7%). Figure 2C presents the sensitivity analysis of this regression with respect to THR (coefficient 0.0234; 95% CI − 0.0064,0.0531; *p* = 0.1230 (to 4 d.p.); I^2^ = 27.3%) and TKR subgroups (coefficient 0.0151; 95% CI − 0.0135,0.0437; *p* = 0.2996 (to 4 d.p.); I^2^ = 96.6%]. Applying Clogg’s method, there was no significant difference between the rate of decreasing postoperative functional gain^53^. The symmetrical funnel plot (Fig. 2D) alleviated concerns discounts publication bias when considering outcome certainty.

Five studies were unsuitable for quantitative synthesis due to outcome reporting^18,20,25,37,38^. Of these three associated decreasing postoperative functional gains with increasing time awaiting surgery^18,20,25^. Apart from Holzapfel et al., all study outcomes were captured 6 to12 months post-operatively. Holzapfel explored the impact of presurgical postponement, e.g., following patient admission for primary elective THR and TKR, over 10 years at a single German centre (N = 10,140) and identified an increased risk of complications and revisions within the postponed surgery group^37^.

Within multivariate regressions of their reported THR (n = 29,303) and TKR (n = 32,602) populations, Nikolova and colleagues identified statistically significant deterioration in 6-month post-operative OHS(0.0951, *p* < 0.01) and OKS(0.0385, *p* < 0.01) per additional day of waiting time, exceeding the estimated effect sizes proposed in this review (Fig. 2C)^30^. This apparent inconsistency between univariate effect and a sufficiently powered multivariate analysis raised concerns around imprecision and unmeasured confounding, and limited certainty in the proposed effect size to “moderate” (Supplementary Table 6). Consequently, it is likely that a negative correlation between preoperative waiting time and postoperative joint function exists.

### Changes in health-related quality of life whilst awaiting total hip or knee replacement surgery

**Figure 3:**
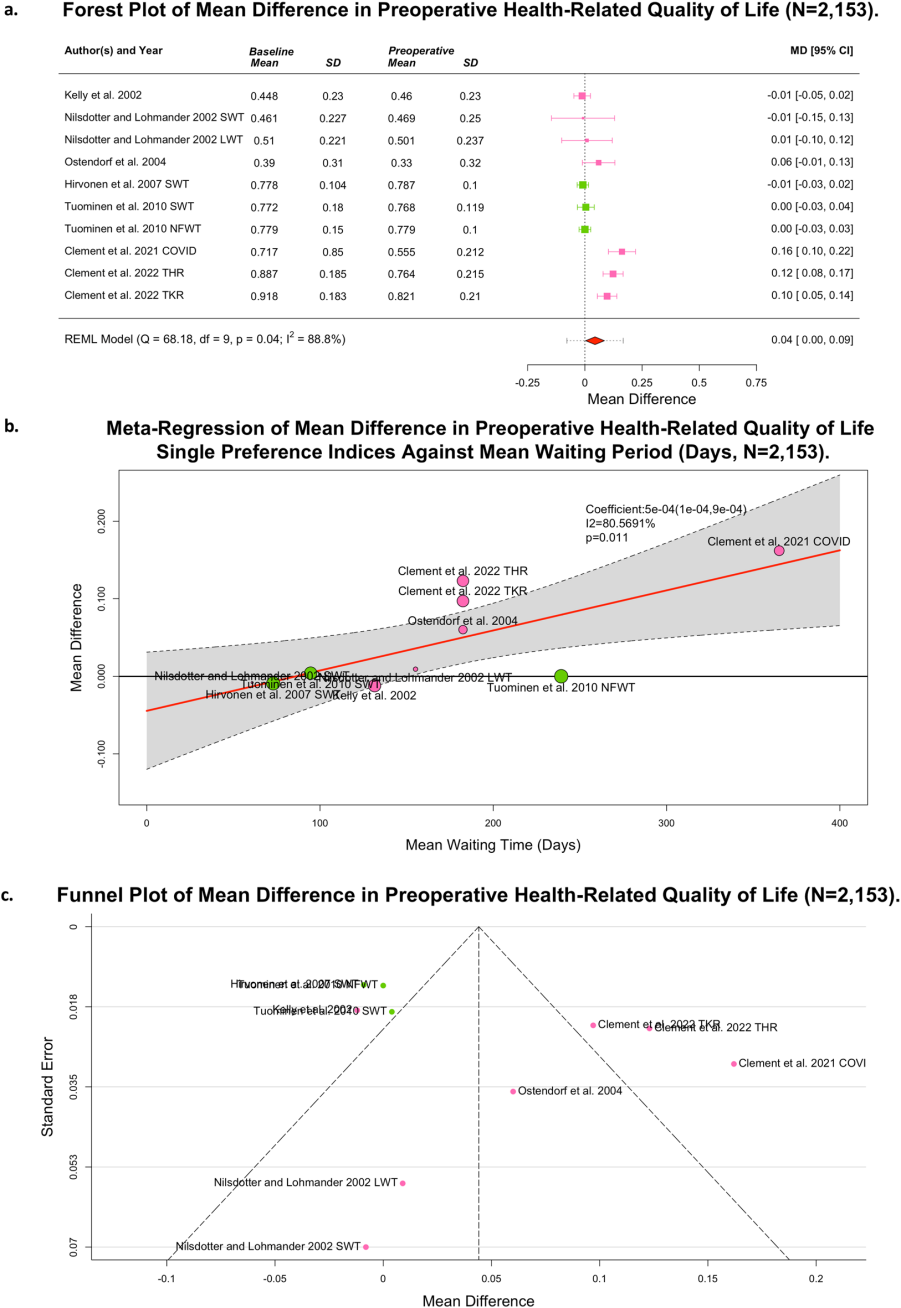
Preoperative Health-Related Quality of Life Outcome Data (N = 2,153]. (**A**) Forest plot of mean difference; (**B**) Meta-regression by mean waiting time; (**C**) Funnel plot. Outcome measures reported include EQ-5D (Pink) and 15D Score (Green].

Eleven studies, representing 7,831 patients commented on changes in HRQOL in the preoperative period^13,16,17,19,22,24,26,27,32,33,35^. Seven of these studies (containing ten cohorts and capturing 2,153 patients) were suitable for meta-analysis^13,16,17,22,27,32,35^. Figure 3A presents a forest plot of the change in single preference-based health related quality of life index associated with waiting for total hip or knee replacement. Whilst awaiting surgery, there was a significant deterioration in patients’ HRQOL (MD 0.04; 95% CI 0.00,0.09; *p* = 0.04; I^2^ = 88.8%) on a scale of 0–1 (worst-to-best). Linear meta-regression (Fig. 3B) showed a statistically significant daily deterioration coefficient of 0.0005 (95% CI − 0.0001.0009; *p* = 0.011 (to 4 d.p.); I^2^ = 80.6%).

Notably, this analysis excluded the non-fixed waiting time cohort of Hivronen et al. Despite 143/183 patients breaking protocol, the reported per-protocol and intention-to-treat admission 15D score and standard deviations were identical to 3 decimal points. In the absence of author clarification, this raised significant concerns around both reporting errors and a negative compliance bias^22^.

Hirvonen et al. reported no statistical difference in 15D score whilst awaiting total hip or knee replacement (MD 0.008; 95% CI 0.002–0.0019; *p* = 0.123), relative to a matched population cohort^19^. McHugh et al. did not report SF-36 subdomains with appropriate granularity to enable quantitative synthesis but identified significant improvements in patient perceptions of the role emotion and general health outcomes (8.5; 95% CI 1.2,15.9 and 7.9; 95% CI 3.6,12.1, respectively)^24^. Desmeules et al. reported HRQOL using only the SF-36 physical functioning, physical health, and bodily pain subdomains, which could not be converted into a single-preference based index^26,54^. This study reported significant deterioration the in physical function (100-point scale) of patients awaiting hip and knee replacements (MD 4.8; 95% CI 7.2,2.4)^26^. Specifically, this was driven by deteriorations within the 3–6 (MD 4.4; 95% CI 7.6, 1.1), 9–12 (11.3; 95% CI 18.4,4.1), and > 12 month (MD 7.1; 95% CI 12.9, 1.3) waiting time subgroups^26^. Significant deterioration was also seen in the 9–12-month subgroup in physical health (MD 20.6; 95% CI: 35.1,6.1)^26^.

Although publication bias was not of concern, (Fig. 3C), risk of bias, primarily due to limited control of confounding, enabled only moderate certainty in this conclusion (Supplementary Table 6).^16,24,35^. Notably, McHugh et al., the study which contributed to this risk of bias, was not included within the quantitative synthesis, and therefore did not influence the slope of the meta-regression (0.0005; 95% CI − 0.0001,0009; *p* = 0.011 (to 4 d.p.); I^2^ = 79.8%). Thus, this risk of bias is immaterial when considering the certainty of the proposed effect size. In summary, HRQOL deteriorates every day whilst awaiting primary elective THR or TKR surgery.

### Post-operative impact of waiting time on health related quality of life following total hip or knee joint replacement

**Figure 4:**
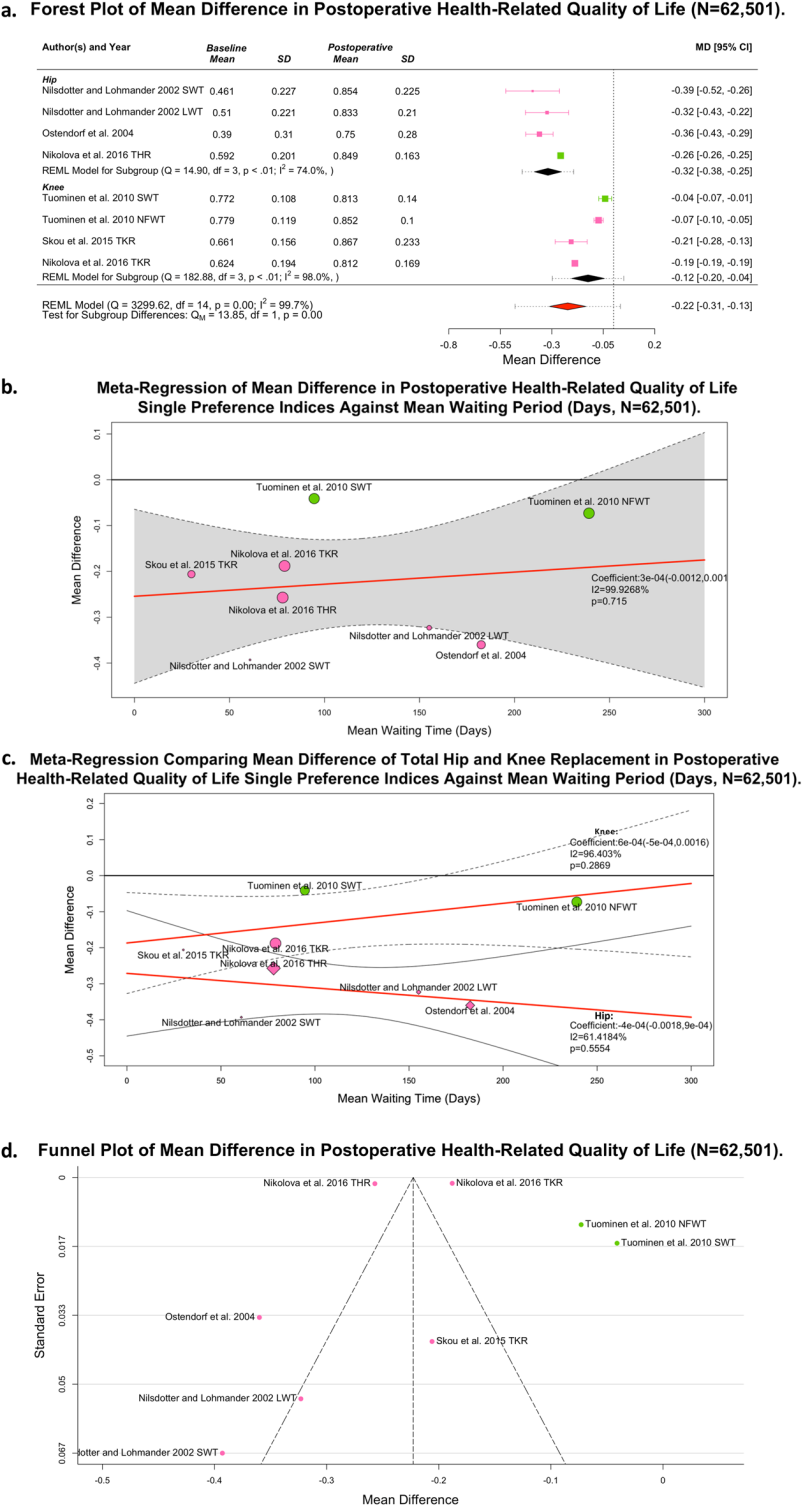
Postoperative Health-Related Quality of Life Outcome Data (n = 62,501). Forest plot of mean difference showing total hip and knee replacement subgroups; B. Pooled meta-regression by mean waiting time; C. Meta-regression sensitivity analysis total hip and knee replacement by mean waiting time. Outcome measures reported include EQ-5D (Pink) and 15D Score (Green].

Seven studies reported postoperative health related quality of life and duration of waiting time, representing 62,777 patients^14,16,18,27–30^. Of these, five studies, containing eight cohorts and representing 62,501 patients, reported single preference index indices of HRQOL(0–1, worst-to-best) and were thus suitable for meta-analysis^16,17,27,29,30^. Figure 4A presents a forest plot of these outcomes, showing an expected significant improvement in long-term HRQOL post-operatively (MD: 0.22; 95% CI 0.13,0.33; *p* = 0.00; I^2^ = 98.0%]. Subgroup analysis identified a significant improvement in HRQOL at long-term follow-up, which again favoured THR (MD: 0.32; 95% CI 0.25,0.38; *p* < 0.01; I^2^ = 74.0%) relative to TKR (MD: 0.12; 95% CI 0.04,0.20; *p* < 0.01; I^2^ = 98.0%) patients.

To explore heterogeneity with regards to presurgical waiting time, Fig. 4B presents a linear meta-regression, identifying a coefficient for daily reduction in post-operative HRQOL of 0.0003 (95% CI − 0.0012,0.0017; *p* = 0.715 (to 4 d.p.); I^2^ = 99.9%]) Fig. 4C compares the influence of type of joint replacement on this. Both TKR (coefficient: 0.0006; 95% CI − 0.0005,0.0016; *p* = 0.287 (to 4 d.p.); I^2^ = 96.4%) and THR (coefficient: − 0.0004; 95% CI − 0.0018,0.0009; p = 0.5554 (to 4 d.p.); I^2^ = 61.4%) had no significant deterioration with waiting time. Clogg’s method did not identify significant differences between these slopes^53^.

Three studies lacked reported parameters necessary for quantitative synthesis^14,18,28^. Using logistic regression, Fielden et al. did not observe a relationship between patients waiting longer than 6 months and EQ-5D^18^. Although commenting on postoperative SF-36, Mahon et al. did not present the subscale data within the article text (there was no accompanying supplementary materials and the authors could not be contacted) precluding extraction^14^. Desmeules et al. reported a significant reduction in the 6-month postoperative SF-36 role physical domain within a subgroup of patients waiting longer than 9 months for TKR, relative to the 3–6 and 6–9 month subgroups, with no statistically significant changes in the additional reported SF-36 subdomains (physical functioning and bodily pain only)^28^.

Six-months postoperatively, Nikolova et al. identified significant EQ-5D reductions of 0.0620 (n = 29,303) and 0.0587 (n = 32,602) per additional day spent waiting for THR and TKR, respectively (rescaled 0–100, worst-to-best)^30^. Subsequently, a post-hoc sensitivity analysis of Nikolova et al. demonstrated negligible influence on the results of the postoperative HRQOL synthesis (N = 596; MD 0.0003; 95% CI − 0.0015,0.0022; *p* = 0.7437 (to 4d.p.); I^2^ = 96.0%). Although the magnitude of Nikolova’s proposed multivariate effect size was consistent with the estimated effect (Fig. 4C) for TKR, the THR outcome showed marked discrepancy with this synthesis. This presented a challenge to certainty when considering this analysis’s univariate meta-regression, particularly around unmeasured confounding within long-term, post-operative outcomes (Supplementary Table 6). As such, whilst THR patients reported greater improvements in HRQOL at 6–12 months post-operatively relative to TKR, there is likely an additional relationship with presurgical waiting time that could not be observed in this analysis. The influence of publication bias, as assessed by funnel plot, (Fig. 4D) was neglibile.

### Patient perspectives on delayed elective total hip or knee replacement surgery

Only five studies that reported patients’ qualitative responses to and perceptions of delayed THR or TKR were identified, summarised in Table 4 (below). All studies (3/3) commenting on psychological responses reported increased patient anxiety, whilst two studies (2/2) reported worsening patient perspectives of both THR and TKR services.

However, it was noted that there were several limitations to this outcome. Firstly, the qualitative nature of these outcomes introduced diverse outcome measures, limited both the directness and comparability of the outcome synthesis. Consequently, this precluded quantitative synthesis and assessment of publication bias, reducing certainty in this outcome to “moderate”. Despite these, all reported outcomes captured negative patient perspectives on delaying THR and TKR for patients, speaking to an underlying deleterious patient experience associated with delayed surgical care.

## Discussion

### Summary of findings

This review presents a contemporary and high-quality (Oxford Centre for Evidence-Based Medicine, OCEBM, Level 1) analysis exploring the impact of presurgical waiting time on primary, elective THR and TKR outcomes^55^. In this systematic review and meta-analysis a significant association between increased presurgical waiting time and deteriorations in patient joint-related function and HRQOL for primary elective THR and TKR were identified. Given reported thresholds, this synthesis estimates that clinically meaningful deterioration (i.e., more than the minimum clinically important difference) in joint specific outcomes and HRQOL likely occur within 6 months of waiting for surgery^32,56,57^. Furthermore, qualitative synthesis indicated that pre-surgical waiting time may deleteriously influence clinical outcomes up to 12-months post-operatively. In alignment with current evidence, post-operative meta-analysis identified a decreased gain in joint specific outcomes and HRQOL at 6 to 12 months post-operatively for primary elective TKR patients, relative to THR^58^. Patients also indicated strongly deleterious perspectives on prolonged presurgical wait for these procedures.

### Limitations

There are several considerations when interpreting this review. Firstly, limitations within the reported literature prevented the use of gold-standard individual patient meta-analysis and/or multivariate meta-regression approaches.

Secondly, the outcome measures synthesised for the primary outcomes of this meta-analysis were diverse. However, only validated outcome measures were utilised in quantitative analysis, and recommended approaches to comparing these were utilised to preserve the validity of this review. Interestingly, there was no trend in preference for particularly outcome measures over time, indicating broad consensus on the utility and validity of each system.

Furthermore, 61,905 of the 89,996 patients reported were drawn from one study^30^. Although this study had a low risk of bias and, through its own multivariate regression, estimated a greater effect size in post-operative joint-function and HRQOL outcomes than was observed in the current meta-analysis, it is possible that this review was underpowered to directly observe any relationship between pre-surgical waiting time and postoperative joint function and HRQOL^30^. Consequently, a deleterious relationship remains plausible. Reassuringly, this meta-analysis independently replicated previous findings in literature, showing a greater improvement in joint functionality and HRQOL following THR relative to TKR in patients 6–12 months post-operatively^58^. Despite this, these postoperative outcomes also likely carried greater exposure to confounding due to the multifactorial nature of postoperative recovery (e.g., when considering the impact of independent predictors of postoperative outcomes, such as capacity for self-care, comorbid diseases (e.g. diabetes), and exercise)^59,60^.

Finally, all reported surgeries were performed in Europe, North America, or New Zealand. This limits the applicability and generalisability of these findings to African, Asian, and South American patient populations^61–63^. Indeed, future research should seek to expand this evidence-base for patients in underserviced health systems. However, when considering the influence of patient heterogeneity and confounding on the reported analyses, both these geographical restrictions and the broader homogeneity of included patient populations serve to mitigate against unmeasurable confounders, (e.g., socioeconomic circumstance), and preserve the internal validity of this work. In alignment with this, whilst included studies capture a 21-year study period with consequent practice variability, reported effect sizes show broad temporal agreement. Interestingly, the significant reduction in preoperative HRQOL associated with prolonged pre-surgical wait time was driven by more recent studies, perhaps indicating the influence of additional risk factors and multimorbidity within modern patient populations.

## Conclusions

This is the first meta-analysis and systematic review to explore both post-operative outcomes and patients’ perspectives to delayed primary elective THR and TKR surgeries. Despite variable outcome assessment, patient voice was unanimous that delays to surgery carried deleterious impacts on both their psychosocial wellbeing and perceptions of care. Indeed, out with this context this finding is reflected within broader elective orthopaedic literature^39,42,44^. Importantly given its influence on HRQOL, future work should seek to further explore patient perspectives on delayed surgical care provision.

The findings of this review differ from previous systematic reviews and meta-analyses on the effect of waiting for THR or TKR. When considering previous reviews, this reported population (preoperative outcomes N = 9,020) exceeds both previous sample sizes (N = 1,646 and N = 2,490 patients for Hoogeboom and Patten, respectively), enhancing the relative power of this meta-analysis(10,11]. Given this larger patient population, and coupled with this review’s implementation of meta-regression to prevent false negative errors from arising around binary discrimination, the pre-operative arm of this analysis is likely powered.

Whilst this meta-analysis clearly shows the deleterious effect of prolonging pre-surgical waiting time in primary elective THR and TKR, no explicit relationship between waiting time and postoperative HRQOL and joint functionality was identified. However, as pre-surgical wait times continue to increase, and patient pre-operative joint function (itself an independent predicator for postoperative joint function) continues to deteriorate, this effect may become more apparent in future^25,45^.

In conclusion, this systematic review and meta-analysis of 89,996 patients undergoing primary elective THR and TKR demonstrates a significant association between prolonged pre-surgical waiting and deleterious preoperative joint-specific outcomes and HRQOL. Furthermore, patient voice unanimously condemned delayed care. Postoperatively, there was a plausible relationship between waiting time and 12-month joint function and HRQOL, whilst patients undergoing THR experience greater joint functionality and HRQOL gains from surgery, relative to TKR. Future work should elucidate the determinants of post-operative joint function and HRQOL following primary THR and TKR, across the global economic spectrum. However, in lieu of this, urgent action should be taken to minimise the ongoing deterioration of patients’ joint functionality, HRQOL, and psychological status whilst awaiting elective primary THR/TKR.

## Methods

### Protocol registration and ethics

The study protocol was registered on the International Prospective Register of Systematic Reviews (PROSPERO) database on 01/03/2022 (CRD42022288128) and was undertaken in accordance with the Preferred Reporting Items for Systematic Review and Meta-analyses (PRISMA, 2020) and Cochrane Handbook for Systematic Reviews of Interventions (2021)^64,65^. Ethical approval was not required.

### Information sources and search strategy

Reported populations of patients undergoing elective primary THRs or TKRs were interrogated, to explore how the duration of the pre-surgical waiting period, defined as the period from placement on waiting list until surgery, influenced joint-specific outcome scores, indices of health-related quality of life, and psychological responses. On 30/1/2023, this search strategy (Supplementary Table 1) was executed on the MEDLINE(R) and In-Process, In-Data-Review & Other Non-Indexed Citations 1946 to January 30th, 2023”, “EMBASE 1980 to 2023 Week 5″, PubMed, and Cochrane CENTRAL databases. No limitations were placed these searches.

### Selection and eligibility criteria

Primary published observational and randomised-controlled literature reporting elective primary TKR and THR populations, their time-to-treatment on presurgical waiting lists, and inclusion of relevant outcome data were eligible. The primary outcomes of this systematic review were validated measures of joint specific outcomes, HRQOL, or psychosocial perspective, with qualitative data being sought secondarily. Individual case-studies and unpublished data were ineligible. To ensure internal validity and preserve external validity, reported populations containing patients aged 16 or under and those undergoing non-elective (e.g., unplanned trauma surgery), partial, or secondary revision of THR/ TKR were also excluded.

Following execution of the search strategy, identified records were de-duplicated and independently screened by two authors to assess relevance. Inter-rater reliability was thus assessed using Cohen’s kappa and disagreements resolved by the senior author^12,65^. Subsequently, relevant abstracts were reviewed to establish final eligibility. All relevant records were reviewed to identify further references of interest. Non-English language manuscripts were only to be excluded after attempting to contact the corresponding authors.

### Data collection and analysis

Following identification, primary outcome data was extracted by the primary author under supervision from the final author. This data was then analysed in prespecified pre- and post-operative subgroups to prevent confounding. Where duplicate data was reported in literature, the revised analysis was retained. Preferred primary outcome measures included clinically validated joint-specific outcome scores (Oxford Hip and Knee scores (OHS, OKS), health-related quality of life scores (HRQOL, e.g., Short Form 36 (SF-36) and EuroQol-5D (EQ5D)), for meta-analysis. However, qualitative, subjective, non-validated, or otherwise incompatible outcome measures were also recorded and manually tabulated for systematic review.

Potential sources of ascertainment bias and heterogeneity were identified through further collection of descriptive datasets (e.g., patient eligibility criteria, duration of presurgical waiting time, study period, study location(s), sample sizes, proportion female sex, and mean patient age was sought for reported populations]. Where missing data was identified or further information required for analysis, clarification was sought from the relevant corresponding author.

Given the diverse outcome measures within scoping literature, comparable data was transformed and represented as mean and standard deviation to facilitate quantitative synthesis. Joint-specific pain and function scores were converted into a common weighted score (0–100, worst-to-best of pain and function subdomains)^65^. Similarly, SF-36 HRQOL (0–100 scale) was converted into a single preference-based EQ-5D index (0–1, worst-to-best) following Ara and Brazier’s method^54^. Where necessary, the standard deviation was imputed using Wan et al.’s adaptive method^66^. Effect sizes were summarised as either mean difference (MD, percentage change) or odds ratios (OR), where possible.

In the absence of individual patient data, summary meta-analysis was undertaken using a random effect residual maximum likelihood model of mean difference. 95% confidence interval (95% CI), and heterogeneity (I^2^) were also calculated. Linear meta-regressions were performed to explore heterogeneity due to variable reported mean pre-surgical waiting times, whilst sensitivity analyses of regression curves were performed using Clogg’s method to enable comparison of postoperative hip and knee arthroplasty data^53,65^. Mixed model meta-analyses and meta-regressions were performed using the “Metafor” R software package^67,68^. Publication bias of quantitative syntheses was assessed using Egger’s funnel plot tests^65,68,69^.

### Risk of bias and certainty assessment

Risk of bias from randomised, non-randomised, and cross-sectional studies following gold-standard Risk of Bias 2 (RoB2), Risk of Bias in Non-Randomised Studies (ROBINS-I), and Joanna Briggs Institute (JBI) frameworks, respectively^47,48,70^. In each instance, the overall risk of bias for sought outcomes was ascribed following the highest constituent domain. Sensitivity analysis was indicated following identification of maximal overall risk of bias. The Grading of Recommendations Assessment, Development and Evaluation (GRADE) framework was also used to assess the certainty of evidence for each outcome synthesised as either “high”, “moderate”, “low” or “very low”^50^.

### Supplementary Information

Supplementary Information.

## Data Availability

Study protocol is publicly accessible on PROPSERO (CRD42022288128]. Search methodology, excluded articles, risk of bias assessments, certainty assessments are included within supplementary materials. Access to included studies is subject to third party restrictions on accessing academic literature. Please direct any queries or data access requests to the corresponding author (GMC].
