# Supplementary material for: The functional and psychological impact of delayed hip and knee arthroplasty: a systematic review and meta-analysis of 89,996 patients
Source: Sci Rep. 2024 Apr 5;14:8032. doi: 10.1038/s41598-024-58050-6 (PMC10997604; doi:10.1038/s41598-024-58050-6)
Supplement: Supplementary file 1 — Supplementary Information. [file 41598_2024_58050_MOESM1_ESM.docx]

**Supplementary Material:**

**Supplementary Figure 1: Preferred Reported Items for Systematic Review and Meta-Analyses Flow Diagram:**

**
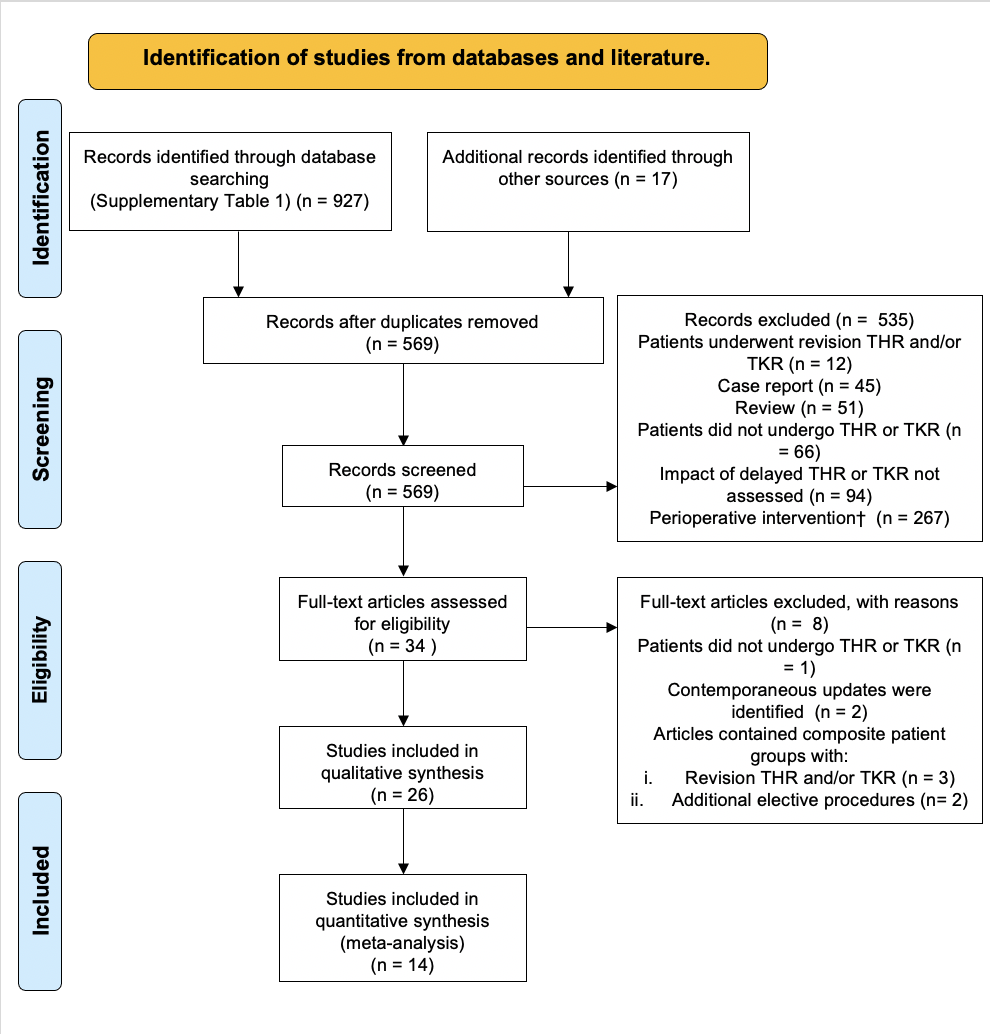
**

**Figure 1:**A Preferred Reported Items for Systematic Review and Meta-Analyses Flow Diagram. This diagram summarises the selection of studies for systematic review and meta-analysis. †Perioperative interventions include orthopaedic implants and techniques and perioperative medications including anaesthetic agent; THR: Total Hip Replacement; TKR: Total Knee Replacement.

**Supplementary Table 1: Search Strategies and Databases Queried**

| # | Search Term | Search 1 & Search 2 | Search 3 | Search 4 |
| --- | --- | --- | --- | --- |
| 1 | Arthroplasty, Replacement, Knee/ | 35609 | 34355 | 0 |
| 2 | Arthroplasty, Replacement, Hip/ | 38029 | 38516 | 0 |
| 3 | “Total Knee Replacement” OR “TKR” OR “Total Knee Arthroplasty” OR “TKA” | 74351 | 30904 | 0 |
| 4 | “Total Hip Replacement” OR “THR” OR “Total Hip Arthroplasty” OR “THA” | 130976 | 60399 | 0 |
| 5 | OR/1-4 | 129261 | 106839 | 0 |
| 6 | Elective Surgical Procedures/ | 3104 | 28409 | 0 |
| 7 | “Elective” | 241349 | 94624 | 0 |
| 8 | 6 OR 7 | 241349 | 94624 | 0 |
| 9 | Time-To-Treatment/ | 33381 | 122431 | 0 |
| 10 | Delay$ or "Delayed Treatment" OR "Delayed Surgery" OR "Delayed Procedure" | 1395561 | 628247 | 0 |
| 11 | Waiting?List OR “Waiting-List” | 25908 | 21894 | 0 |
| 12 | OR/9-11 | 1445402 | 1704094 | 0 |
| 13 | AND/5,8,12 | 438 | 450 | 0 |

**Supplementary Table 1:**  Summary of literature searches performed. Search 1: MEDLINE(R) and In-Process, In-Data-Review & Other Non-Indexed Citations 1946 to January 30th, 2023; Search 2: EMBASE 1980 to 2023 Week 5; Search 3: PUBMED 30/01/2023; and Search 4: CENTRAL 30/01/2023.

**Supplementary Table 2: Studies Excluded at Full-Text Screening**

| Authors | Study Design | Study Setting | | | | Study Population in Analysis | | | | Exclusion |
| --- | --- | --- | --- | --- | --- | --- | --- | --- | --- | --- |
|  |  | Eligibility Criteria | Study Period | Country | Number of Centres | Exposure (Mean Waiting Time, Days) | Sample Size  [Exposure Groups] | Mean Age (Years) [Exposure Groups] | Sex (% Female) [Exposure Groups] |  |
| Derret et al. 1999^1^ | Cross-Sectional | Patients awaiting non-urgent hip or knee replacement on 5^th^ October 1995 | 1995 | New Zealand | Single-Centre | 0-182.5, 182.5-365, 365-730, >730 | 47 [N/A] | 65-69* [N/A] | 60% [N/A] | Unspecified primary or THR/ TKR replacement surgery in analysis. |
| Tuominen et al. 2009^2^ | RCT | Patients awaiting primary TKR for OA >16 years-old. | 2002-2003 | Finland | 3 Hospitals | 74, 194 | 145, 175 | 65 [64, 66] | 49.4% [49.2%, 52.3%] | Cohort re-reported and analysis superseded by Tuominen et al. 2010 (Included). |
| Brown et al. 2020^3^ | Cross-Sectional | Patients scheduled for primary THR/ TKR cancelled because of COVID-19. | 2020 | USA | 7 Centres | N/A- COVID-19 Pandemic | 360 | 65 | 58% | Expanded cohort updated in Brown et al. 2021 (Included). |
| Wilson et al. 2020^4^ | Cross-Sectional | Patients scheduled for primary THR/ TKR cancelled because of COVID-19. | 2020 | USA | Single-Centre | N/A- COVID-19 Pandemic | 111 | 65 | 65% | Included revision arthroplasties in analysis. |
| Madanipour et al. 2020^5^ | Cross-Sectional | Patients awaiting hip or knee arthroplasty. | 2020 | UK | Single-Centre | N/A- COVID-19 Pandemic | 136 | 68.5 | 36.5% | Included revision and unicompartmental arthroplasties in analysis. |
| Knebel et al. 2021^6^ | Cross-Sectional | Patients awaiting elective orthopaedic procedure cancelled due t COVID-19. | 2020 | Germany | Single-Centre | N/A- COVID-19 Pandemic | 119 | 60-69* | 44% | Included revision THR, revision TKR, arthroscopy and biopsy, foot and ankle procedures in analysis. |
| Farrow et al. 2022^7^ | Retrospective Cohort | Patients undergoing primary elective THR/ TKR/ UKR. | 2017-2021 | UK | Single-Centre | N/A- Not Collected. Participants stratified by preoperative joint function [low, high]. | 513 [244,269] | 69.4 | 57.7% | No waiting time data available.  Included UKR in analysis. |
| Mandl et al. 2020^8^ | Prospective Cohort | Patients with knee OA undergoing TKR. | 2020-2021 | USA | Single-Centre | N/A- Positive COVID-19 immunoglobulins [positive, negative] | 144 [67,77] | 64 | 58.3% | No waiting time data available. |

***Supplementary Table 2:*** *Summary characteristics and exclusory cases of studies excluded in systematic review and meta-analysis.* *RCT*: *Randomised Controlled Trial; THR: Total Hip Replacement; TKR: Total Knee Replacement; OA: Osteoartritis; UK: United Kingdom; USA: United States of America; UKR: Unicompartmental Knee Replacement *Median value*

**Supplementary Table 3: Risk of Bias in Randomised Controlled Trials by Risk of Bias 2 (ROB-2).**

| Study Name | Risk of bias arising from the randomisation process | Risk of bias due to deviations from the intended intervention | Risk of bias due to missing outcome data | Risk of bias in measurement of the outcome | | | Risk of bias in selection of reported result | | | Overall Risk of Bias | | |
| --- | --- | --- | --- | --- | --- | --- | --- | --- | --- | --- | --- | --- |
|  |  |  |  | A | B | C | A | B | C | A | B | C |
| Hirvonen et al. 2007^9^ | ↓ | - | ↓ | ↓ | ↓ | X | ↓ | ↓ | X | - | - | X |
| Tuominen et al. 2010^10^ | ↓ | - | ↓ | ↓ | ↓ | X | ↓ | ↓ | X | - | - | X |
| Skou et al. 2022^11^ | ↓ | - | ↓ | ↓ | ↓ | X | ↓ | ↓ | X | - | - | X |

***Supplementary Table 3:*** *Risk of bias in randomised studies was considered within the ROB-2 framework*^12^*. For each domain, risk was assessed as either not applicable (N/A), insufficient information to assess (N/I), increased, decreased (↓), or unchanged (-) risk. Overall risk of bias was attributed by the highest reported risk in any domain.* *Outcomes reviewed: A: Hip or knee joint-specific function; B: Global Health-Related Quality of Life indices; C: patient perspectives. .*

**Supplementary Table 4: Risk of Bias in Cohort and Case-Control Studies by Risk of Bias in Non-Randomised Studies (ROBINS-I).**

| Article | Risk of Bias Corresponding to: | | | | | | | | | | | | | Overall Risk of Bias in Design: | | |
| --- | --- | --- | --- | --- | --- | --- | --- | --- | --- | --- | --- | --- | --- | --- | --- | --- |
|  | Control of Confounding | Selection of Participants | Classification of Intervention | Deviation from Intervention | Missing Data | | | Outcome Measurement | | | Selection of Reported Result | | |  |  |  |
|  |  |  |  |  | A | B | C | A | B | C | A | B | C | A | B | C |
| Kelly et al. 2001^13^ | 2 | 1 | 1 | N/A | 1 | 1 | X | 1 | 1 | X | 2 | 2 | X | 2 | 2 | X |
| Hajat et al. 2002^14^ | 2 | 1 | 1 | N/A | 1 | X | X | 1 | X | X | 2 | X | X | 2 | X | X |
| Mahon et al. 2002^15^ | 3 | 1 | 1 | N/A | 1 | 1 | 1 | 1 | 1 | 1 | 2 | 2 | 2 | 3 | 3 | 3 |
| Nilsdotter and Lohmander 2002^16^ | 3 | 2 | 1 | N/A | X | 1 | X | 1 | 1 | X | 2 | 2 | X | 3 | 3 | X |
| Ostendorf et al. 2004^17^ | 2 | 1 | 1 | N/A | 1 | 1 | X | 1 | 1 | X | 2 | 2 | X | 2 | 2 | X |
| Fielden et al. 2005^18^ | 2 | 1 | 1 | N/A | 1 | 1 | X | 1 | 1 | X | 2 | 2 | X | 2 | 2 | X |
| Garbuz et al. 2006^19^ | 2 | 1 | 1 | N/A | 2 | X | X | 1 | X | X | 2 | X | X | 2 | X | X |
| Hirvonen et al. 2006^9^ | 1 | 1 | 1 | N/A | X | 2 | X | X | 1 | X | X | 1 | X | X | 2 | X |
| Ahmad and Konduru 2007^20^ | 3 | 1 | 1 | N/A | 1 | X | X | 1 | X | X | 2 | X | X | 3 | X | X |
| Kapstad et al. 2007^21^ | 2 | 1 | 1 | N/A | 3 | X | X | 1 | X | X | 2 | X | X | 3 | X | X |
| McHugh et al. 2008^22^ | 3 | 1 | 1 | N/A | 2 | 2 | X | 1 | 1 | X | 2 | 2 | X | 3 | 3 | X |
| Escobar et al. 2009^23^ | 3 | 1 | 1 | N/A | 2 | X | X | 1 | X | X | 2 | X | X | 3 | X | X |
| Desmeules et al. 2010^24^ | 2 | 1 | 1 | N/A | 3 | 3 | X | 1 | 1 | X | 1 | 1 | X | 3 | 3 | X |
| Desmeules et al. 2012^25^ | 2 | 1 | 1 | N/A | 3 | 3 | X | 1 | 1 | X | 1 | 1 | X | 3 | 3 | X |
| Nikolova et al. 2016^26^ | 2 | 1 | 1 | N/A | 1 | 1 | X | 1 | 1 | X | 1 | 1 | X | 1 | 1 | X |
| Clement et al. 2021^27^ | 1 | 1 | 1 | N/A | X | 1 | 1 | 1 | 1 | N/I | X | 1 | 1 | X | 1 | 2 |
| Farrow et al. 2021^28^ | 2 | 2 | 1 | N/A | 1 | X | X | 1 | X | X | 2 | X | X | X | X | 2 |
| Holzapfel et al. 2022^29^ | 1 | 1 | 2 | N/A | 1 | X | X | 1 | X | X | 2 | X | X | 2 | X | X |
| Morri et al. 2022^30^ | 1 | 1 | 1 | N/A | 1 | X | X | 1 | X | X | 1 | X | X | 1 | X | X |

***Supplementary Table 4:*** *Risk of bias in non-randomised studies was considered according to the ROBINS-I framework*^31^*. Each domain was assessed as either not applicable (N/A) insufficient information to assess (N/I), low (1), moderate (2), serious (3) or critical risk of bias(4). Overall risk of bias was attributed by the highest reported risk in any domain. Outcomes reviewed: A: Hip or knee joint-specific function; B: Global Health-Related Quality of Life indices; C: patient perspectives.*

**Supplementary Table 5: Risk of Bias in Cross-Sectional Studies by Joanna Briggs Institute (JBI) Cross-Sectional Study Framework.**

| Article | Sources of Bias: | | | | | | | | | | | | Overall Risk of Bias: | | |
| --- | --- | --- | --- | --- | --- | --- | --- | --- | --- | --- | --- | --- | --- | --- | --- |
|  | Clear Eligibility Criteria. | Clear  Study Subjects and  Setting. | Valid Measurement of Exposure. | Standardised Criteria | Sources of Confounding Identified. | Control of Identified Con-founding | Valid Measurement of Outcome | | | Appropriate Statistical Analysis. | | |  |  |  |
|  |  |  |  |  |  |  | A | B | C | A | B | C | A | B | C |
| Brown et al. 2021^32^ | Y | Y | X | Y | Y | Y | N | X | N | Y | X | Y | 2 | X | 2 |
| Johnson et al. 2021^33^ | Y | Y | X | Y | N | N | N | X | Y | Y | X | Y | 3 | X | 2 |
| Clement et al. 2022^34^ | Y | Y | Y | Y | Y | Y | X | Y | X | X | Y | X | X | 1 | X |
| Grace et al. 2022^35^ | Y | N | X | Y | N | N | X | X | Y | X | X | Y | X | X | 3 |

***Supplementary Table 5:*** *JBI risk of bias assessment in cross-sectional studies*^36^*. Each domain was considered as either insufficient information to assess (N/I), yes (Y), no (N), or not applicable (X). Additionally, overall risk of bias thus determined as low (1), moderate (2), serious (3) or critical risk (4). Outcomes reviewed: A: Hip or knee joint-specific function; B: Global Health-Related Quality of Life indices; C: patient perspectives.*

**Supplementary Table 6: Grading of Recommendations Assessment, Development and Evaluation Certainty of Evidence Assessment (GRADE).**

| Outcome | Risk of Bias | Inconsistency | Indirectness | Imprecision | Publication Bias | Overall Certainty |
| --- | --- | --- | --- | --- | --- | --- |
| Waiting time on preoperative joint-specific functional scores. | Not Serious | Not Serious | Not Serious | Not Serious | Not Serious | High |
| Waiting time on postoperative joint-specific functional scores. | Serious | Serious | Not Serious | Serious | Not Serious | Moderate |
| Waiting time on preoperative health-related quality of life indices. | Serious | Not Serious | Not Serious | Not Serious | Not Serious | Moderate |
| Waiting time on postoperative health-related quality of life indices. | Serious | Very Serious | Not Serious | Serious | Not Serious | Low |
| Waiting time on psychosocial dimension of patient experience. | Serious | Not Serious | Serious | Not Serious | N/A^A^ | Moderate |

***Supplementary Table 6:*** *GRADE Quality of Evidence for synthesised outcomes*^37^*. Outcome certainty started at “High” overall certainty (Green) and could be downgraded to “Moderate” (Green), “Low” (Orange) or “Very Low” (Red) according to the most limited constituent domain: not serious (-0, Green), serious (-1, Yellow) or very serious (-2, Red). There were no grounds to upgrade evidence quality to “High” (Green) certainty of evidence; A: publication biases could also not be assessed in this outcome.*

**References:**

1. Derrett S, Paul C, Morris JM. Waiting for elective surgery: effects on health-related quality of life. Int J Qual Health Care. 1999 Feb;11(1):47–57.

2. Tuominen U, Sintonen H, Hirvonen J, Seitsalo S, Paavolainen P, Lehto M, et al. The effect of waiting time on health and quality of life outcomes and costs of medication in hip replacement patients: a randomized clinical trial. Osteoarthritis and Cartilage. 2009 Sep 1;17(9):1144–50.

3. Brown TS, Bedard NA, Rojas EO, Anthony CA, Schwarzkopf R, Barnes CL, et al. The Effect of the COVID-19 Pandemic on Electively Scheduled Hip and Knee Arthroplasty Patients in the United States. J Arthroplasty. 2020 Jul;35(7S):S49–55.

4. Wilson JM, Schwartz AM, Farley KX, Roberson JR, Bradbury TL, Guild GN. Quantifying the Backlog of Total Hip and Knee Arthroplasty Cases: Predicting the Impact of COVID-19. HSS J. 2020 Nov;16(Suppl 1):85–91.

5. Madanipour S, Al-Obaedi O, Ayub A, Iranpour F, Subramanian P. Resuming elective hip and knee arthroplasty in the COVID-19 era: a unique insight into patient risk aversion and sentiment. Ann R Coll Surg Engl. 2021 Feb;103(2):104–9.

6. Knebel C, Ertl M, Lenze U, Suren C, Dinkel A, Hirschmann MT, et al. COVID-19-related cancellation of elective orthopaedic surgery caused increased pain and psychosocial distress levels. Knee Surg Sports Traumatol Arthrosc. 2021;29(8):2379–85.

7. Farrow L, Redmore J, Talukdar P, Clement N, Ashcroft GP. Prioritisation of patients awaiting hip and knee arthroplasty: Lower pre-operative EQ-5D is associated with greater improvement in quality of life and joint function. Musculoskeletal Care. 2022;20(4):892–8.

8. Lisa Mandl, Benjamin Swett, Robyn Lipschultz, Myriam Lin, Alison Zhao, Carola Hanreich, Dongmei Sun, Kethy Jules-elysee and Friedrich Boettner. Is SARS-CoV-2 Associated with Worse Outcomes After Total Knee Arthroplasty? In: ACR Meeting Abstracts [Internet]. Philadelphia, PA.; 2022 [cited 2023 Mar 22]. Available from: https://acrabstracts.org/abstract/is-sars-cov-2-associated-with-worse-outcomes-after-total-knee-arthroplasty/

9. Hirvonen J, Blom M, Tuominen U, Seitsalo S, Lehto M, Paavolainen P, et al. Evaluating waiting time effect on health outcomes at admission: a prospective randomized study on patients with osteoarthritis of the knee joint. J Eval Clin Pract. 2007 Oct;13(5):728–33.

10. Tuominen U, Sintonen H, Hirvonen J, Seitsalo S, Paavolainen P, Lehto M, et al. Is longer waiting time for total knee replacement associated with health outcomes and medication costs? Randomized clinical trial. Value Health. 2010 Dec;13(8):998–1004.

11. Skou ST, Roos EM, Laursen MB, Rathleff MS, Arendt-Nielsen L, Simonsen O, et al. A Randomized, Controlled Trial of Total Knee Replacement. N Engl J Med. 2015 Oct 22;373(17):1597–606.

12. Sterne JAC, Savović J, Page MJ, Elbers RG, Blencowe NS, Boutron I, et al. RoB 2: a revised tool for assessing risk of bias in randomised trials. BMJ. 2019 Aug 28;366:l4898.

13. Kelly KD, Voaklander DC, Johnston DWC, Newman SC, Suarez-Almazor ME. Change in pain and function while waiting for major joint arthroplasty. The Journal of Arthroplasty. 2001 Apr 1;16(3):351–9.

14. Hajat S, Fitzpatrick R, Morris R, Reeves B, Rigge M, Williams O, et al. Does waiting for total hip replacement matter? Prospective cohort study. J Health Serv Res Policy. 2002 Jan;7(1):19–25.

15. Mahon JL, Bourne RB, Rorabeck CH, Feeny DH, Stitt L, Webster-Bogaert S. Health-related quality of life and mobility of patients awaiting elective total hip arthroplasty: a prospective study. CMAJ. 2002 Nov 12;167(10):1115–21.

16. Nilsdotter A ‐K., Lohmander LS. Age and waiting time as predictors of outcome after total hip replacement for osteoarthritis. Rheumatology. 2002 Nov 1;41(11):1261–7.

17. Ostendorf M, Buskens E, van Stel H, Schrijvers A, Marting L, Dhert W, et al. Waiting for total hip arthroplasty: avoidable loss in quality time and preventable deterioration. J Arthroplasty. 2004 Apr;19(3):302–9.

18. Fielden JM, Cumming JM, Horne JG, Devane PA, Slack A, Gallagher LM. Waiting for hip arthroplasty: economic costs and health outcomes. J Arthroplasty. 2005 Dec;20(8):990–7.

19. Garbuz DS, Xu M, Duncan CP, Masri BA, Sobolev B. Delays worsen quality of life outcome of primary total hip arthroplasty. Clin Orthop Relat Res. 2006 Jun;447:79–84.

20. Ahmad I, Konduru S. Change in functional status of patients whilst awaiting prinmary total knee arthroplasty. Surgeon. 2007 Oct;5(5):266–7.

21. Kapstad H, Rustøen T, Hanestad BR, Moum T, Langeland N, Stavem K. Changes in pain, stiffness and physical function in patients with osteoarthritis waiting for hip or knee joint replacement surgery. Osteoarthritis and Cartilage. 2007 Jul 1;15(7):837–43.

22. McHugh GA, Luker KA, Campbell M, Kay PR, Silman AJ. Pain, physical functioning and quality of life of individuals awaiting total joint replacement: a longitudinal study. J Eval Clin Pract. 2008 Feb;14(1):19–26.

23. Escobar A, Quintana JM, González M, Bilbao A, Ibañez B. Waiting list management: priority criteria or first-in first-out? A case for total joint replacement. J Eval Clin Pract. 2009 Aug;15(4):595–601.

24. Desmeules F, Dionne CE, Belzile E, Bourbonnais R, Frémont P. The burden of wait for knee replacement surgery: effects on pain, function and health-related quality of life at the time of surgery. Rheumatology (Oxford). 2010 May;49(5):945–54.

25. Desmeules F, Dionne CE, Belzile ÉL, Bourbonnais R, Frémont P. The impacts of pre-surgery wait for total knee replacement on pain, function and health-related quality of life six months after surgery. J Eval Clin Pract. 2012 Feb;18(1):111–20.

26. Nikolova S, Harrison M, Sutton M. The Impact of Waiting Time on Health Gains from Surgery: Evidence from a National Patient-reported Outcome Dataset. Health Econ. 2016 Aug;25(8):955–68.

27. Clement ND, Scott CEH, Murray JRD, Howie CR, Deehan DJ. The number of patients “worse than death” while waiting for a hip or knee arthroplasty has nearly doubled during the COVID-19 pandemic. The Bone & Joint Journal. 2021 Apr;103-B(4):672–80.

28. Farrow L, Gardner WT, Tang CC, Low R, Forget P, Ashcroft GP. Impact of COVID-19 on opioid use in those awaiting hip and knee arthroplasty: a retrospective cohort study. BMJ Qual Saf [Internet]. 2021 Sep 12 [cited 2022 Sep 12]; Available from: https://qualitysafety.bmj.com/content/early/2022/06/30/bmjqs-2021-013450

29. Holzapfel DE, Meyer M, Thieme M, Pagano S, von Kunow F, Weber M. Delay of total joint replacement is associated with a higher 90-day revision rate and increased postoperative complications. Arch Orthop Trauma Surg. 2022 Nov 4;1–8.

30. Morri M, Ruisi R, Peccerillo V, Franchini N, Magli AO, Forni C. The impact of the pandemic on functional outcomes for joint replacement patients: An observational study. Medicine. 2022 Sep 9;101(36):e30395.

31. Sterne JA, Hernán MA, Reeves BC, Savović J, Berkman ND, Viswanathan M, et al. ROBINS-I: a tool for assessing risk of bias in non-randomised studies of interventions. BMJ [Internet]. 2016 Oct 12 [cited 2020 Nov 23];355. Available from: https://www.bmj.com/content/355/bmj.i4919

32. Brown TS, Bedard NA, Rojas EO, Anthony CA, Schwarzkopf R, Stambough JB, et al. The Effect of the COVID-19 Pandemic on Hip and Knee Arthroplasty Patients in the United States: A Multicenter Update to the Previous Survey. Arthroplast Today. 2021 Feb;7:268–72.

33. Johnson NR, Odum S, Lastra JD, Fehring KA, Springer BD, Otero JE. Pain and Anxiety due to the COVID-19 Pandemic: A Survey of Patients With Delayed Elective Hip and Knee Arthroplasty. Arthroplast Today. 2021 Aug;10:27–34.

34. Clement ND, Wickramasinghe NR, Bayram JM, Hughes K, Oag E, Heinz N, et al. Significant deterioration in quality of life and increased frailty in patients waiting more than six months for total hip or knee arthroplasty : a cross-sectional multicentre study. Bone Joint J. 2022 Nov;104-B(11):1215–24.

35. Grace TR, Eralp I, Khan IA, Goh GS, Siqueira MB, Austin MS. Are Patients With End-Stage Arthritis Willing to Delay Arthroplasty for Payer-Mandated Physical Therapy? The Journal of Arthroplasty. 2022 Jun 1;37(6, Supplement):S27–31.

36. Sandeep Moola, Zachary Munn, Catalin Tufanaru, Edoardo Aromataris, Kim Sears, Raluca Sfetc, Marian Currie, Karolina Lisy, Rubab Qureshi, Patrick Mattis, Pei-Fan Mu. Chapter 7: Systematic reviews of etiology and risk - JBI Manual for Evidence Synthesis. In: JBI Manual for Evidence Synthesis [Internet]. JBI; 2020 [cited 2022 Jun 19]. Available from: https://doi.org/10.46658/JBIMES-20-08

37. Guyatt GH, Oxman AD, Vist GE, Kunz R, Falck-Ytter Y, Alonso-Coello P, et al. GRADE: an emerging consensus on rating quality of evidence and strength of recommendations. BMJ. 2008 Apr 24;336(7650):924–6.
